# Comparing the gastrointestinal barrier function between growth-retarded and normal yaks on the Qinghai-Tibetan Plateau

**DOI:** 10.7717/peerj.9851

**Published:** 2020-09-03

**Authors:** Jian Ma, Ali Mujtaba Shah, Zhisheng Wang, Rui Hu, Huawei Zou, Xueying Wang, Guang Cao, Quanhui Peng, Bai Xue, Lizhi Wang, Suonan Zhao, Xiangying Kong

**Affiliations:** 1 Low Carbon Breeding Cattle and Safety Production University Key Laboratory of Sichuan Province, Animal Nutrition Institute, Sichuan Agricultural University, Chengdu, China; 2 Haibei Demonstration Zone of Plateau Modern Ecological Animal Husbandry Science and Technology, Haibei, China

**Keywords:** Growth-retarded yak, Ruminal epithelium, Jejunal epithelium, Inflammatory cytokine, Tight junction protein

## Abstract

**Background:**

Yak (*Bos grunniens*) is an ancient bovine species on the Qinghai-Tibetan Plateau. Due to extremely harsh condition in the plateau, the growth retardation of yaks commonly exist, which can reduce the incomes of herdsman. The gastrointestinal barrier function plays a vital role in the absorption of nutrients and healthy growth. Functional deficiencies of the gastrointestinal barrier may be one of the contributors for yaks with growth retardation.

**Methods:**

To this end, we compared the growth performance and gastrointestinal barrier function of growth-retarded (GRY) and normal yaks (GNY) based on average daily gain (ADG), serum parameters, tissue slice, real-time PCR, and western blotting, with eight yaks in each group.

**Results:**

GRY exhibited lower (*P* < 0.05) average daily gain as compared to GNY. The diamine oxidase, D-lactic acid, and lipopolysaccharide concentrations in the serum of GRY were significantly higher (*P* < 0.05) than those of GNY. Compared to GNY, the papillae height in the rumen of GRY exhibited lower (*P* = 0.004). In jejunum, with the exception of higher villus height, width, and surface area in GNY, numerical difference (*P* = 0.61) was detected between two groups for crypt depth. Both in rumen and jejunum, the mRNA expression of interleukin-1beta in GRY was markedly higher (*P* < 0.05) than that in GNY, but an opposite trend was found in interleukin-10 expression. Moreover, GRY showed a higher (*P* < 0.05) tumor necrosis factor-alpha mRNA expression in the rumen. The claudin-1 (CLDN1), occludin (OCLN), and zonula occludens-1 (ZO1) expressions of GRY in rumen and jejunum were significantly down-regulated (*P* < 0.05) as compared to GNY. The correlation analysis identified that in rumen and jejunum, there was a positive correlation between interleukin-10 and CLDN1, OCLN, and ZO1 mRNA expressions, but the tumor necrosis factor-alpha was negatively correlated with CLDN1, OCLN, and ZO1. In the rumen, the ADG was positively correlated with papillae surface area, and a same relationship between ADG and CLDN1, OCLN, and ZO1 expressions was found.

**Conclusion:**

The results indicated that the ruminal and jejunal barrier functions of GRY are disrupted as compared to GNY. In addition, our study provides a potential solution for promoting the growth of GRY by enhancing the gastrointestinal barrier function.

## Introduction

Yaks (*Bos grunniens*) live in extremely harsh condition on the Qinghai-Tibetan Plateau at altitudes from 2,500 m to 6,000 m above sea level. More than 92% of the world’s yaks are distributed in China, and the milk, meat, fur, bone (carving into beads), and fuel (feces for living fuel) of yaks are the major living resources and financial income for the local Tibetan herdsman (Zou et al., 2020). Yaks are used as the transport on the plateau by the herdsman. The meat of yaks is increasingly popular and provides a significant contribution to the local economy. Thus, studies are increasingly focusing on the growth performance of yaks (Hu et al., 2016). Besides, yak acts as an essential ecological niche in the Qinghai-Tibetan Plateau ecosystem (Zhang et al., 2016), and remain semi-domesticated status, herding on the plateau meadow with natural breeding. However, the climate of Qinghai-Tibetan Plateau is severely cold with heavy snowfall in the long frigid season (from October to May with average temperature −15 to −5 °C). Because of snow-covered and withered grass in the cold season, the grassland of the plateau is extremely short of forage. In addition, the reproduction characteristics (the period of pregnancy and delivery are usually in cold season) of yaks lead to severe malnutrition at young age, which may restrict healthy growth of yaks in the future (Zi, 2003). Therefore, it is a common phenomenon that growth-retarded yaks (GRY) exist on the Qinghai-Tibetan Plateau.

The GRY with low body weight (BW) and feed efficiency, high morbidity and mortality can reduce the farming economic income of herdsman. It is an urgent problem to find the underlying factors and promote the compensatory growth of GRY. Our previous study found that compared to growth normal yaks (GNY), the ruminal weight of GRY were lower (Hu et al., 2016). The healthy development of the gastrointestinal tract is fundamental to animals’ body nutrient deposition and growth, and it plays an important role not only in feed digestion and absorption of nutrients, but also in endocrine and immune functions (Celi et al., 2017; Powell, Walker & Talley, 2017). The rumen is an important digestive organ for ruminants to digest roughage, particularly in yaks (digesting natural grass). Besides, previous studies reported that severe malnutrition during early life damages the structure and function of the gastrointestinal tract, and then results in subsequent growth retardation (Yambayamba, Price & Jones, 1996; Meyer & Caton, 2016; Hu et al., 2017).

The intestinal barrier integrity is vital to the absorption of nutrients and health in animals. The tight junction protein (TJP) is the key parameter to evaluate the gastrointestinal barrier function. In addition, the impairment of the gastrointestinal barrier can be evaluated through measuring the contents of diamine oxidase (DAO), D-lactic acid (D-LA), and endotoxin-1 (ET-1) in serum (Zhang et al., 2014; Meng et al., 2016). The disruption of the ruminal epithelial barrier function can up-regulate the mRNA expressions of cytokines and down-regulate the expressions of TJPs (Liu et al., 2013). Other studies reported that the injured epithelial barrier function of jejunum could decrease the TJP expressions (Albin et al., 2007; Zhang et al., 2019d). The stress (e.g., malnutrition) of animals during the early life can weaken the barrier function of the gastrointestinal tract and healthy growth (Smith et al., 2010; Chen et al., 2016). Based on previous studies, we hypothesized that the growth retardation of yaks was mainly caused by the functional deficiencies of the gastrointestinal barrier. Therefore, in this study, we compared the growth performance, serum permeability parameters, gastrointestinal morphology, expressions of cytokines, and TJPs between GRY and GNY. Furthermore, the relationship between cytokines and TJPs was also evaluated.

## Materials and Methods

The animal study was carried out according to the Regulation on the Administration of Laboratory Animals (2017 Revision) promulgated by Decree No. 676 of the State Council. All experimental procedures were authorized and approved by the Institutional Animal Care and Use Committee of Sichuan Agricultural University (Permit No. SYXK-2019-187).

### Experimental animals

The animals were chosen from the grassland (altitude about 3,200 m) of Animal Husbandry and Veterinary Institute of Haibei Prefecture, Qinghai Province, China. The current study was conducted from December (2018) to March (2019). According to the previous study on BW of yak population by our group (Hu et al., 2016) and investigation results of local yak population by the Animal Husbandry and Veterinary Institute of Haibei Prefecture, growth retardation is defined as BW that the BW of one yak with the same breed and age is in the low 10% of yak population (Jones et al., 2012). Eight male Qinghai plateau yaks (74.0 ± 6.41 kg of BW and 480 ± 4.50 days of age) with growth retardation were selected as the GRY group. Another 8 male growth normal yaks (112 ± 4.03 kg of BW and 480 ± 5.00 days of age) with the same breed were used as the GNY group. All animals were pastured on the same grassland without supplementary feed. The main natural grass are *Elymus nutans*, *Kobresia humilis*, *Kobresia pygmaea*, and *Carex moorcroftii* in the pasture. The nutrient levels of the natural grass are presented as follows (dry basis): 5.02% crude protein, 53.1% neutral detergent fiber, 34.3% acid detergent fiber, 2.61% ether extract, and 11.2% crude ash. After the yaks were marked with ear tags, a 7 d adaptation period followed by 60 d of experimental period was implemented.

### Sample collection

The BW of all yaks was measured on d 0 and 60 at 07:30, and the average daily gain (ADG) was calculated via the initial and final BW. On d 60, blood were sampled from all yaks at 08:00 via the jugular vein, then the serum samples were obtained by centrifugation (3,000 × *g* for 15 min, 4 °C) and stored at −80 °C for analysis of DAO, D-LA, ET-1, and lipopolysaccharide (LPS) using ELISA kits (Solarbio Science and Technology, Beijing, China) (Wu et al., 2018; Liu et al., 2013). After fasting of 12 h, 6 yaks of each group that were close to group average BW were slaughtered by captive bolt stunning. The process of slaughter was followed the national standard Operating Procedures (GB/T 19477-2004, cattle slaughtering, China).

After removing the digesta, tissues of rumen and jejunum were collected. In the ventral sac, the ruminal epithelium (1 g) was isolated from the serosal and muscular layers, rinsed with ice-cold sterile phosphate buffered saline (PBS), minced and put into 1.5 mL sterile tubes, then rapidly frozen in liquid nitrogen and stored at −80 °C for quantitative real-time PCR (qRT-PCR) and western blotting (WB) analysis (Wang et al., 2017). Besides, ruminal epithelium (2 cm × 2 cm) was removed from the ventral and dorsal sac. The tissue samples were placed into 4% paraformaldehyde overnight, and then dehydrated and embedded in paraffin. Subsequently, the samples were cut into 3 sections with 5 m thick and stained with Hematoxylin and Eosin for morphological study. The morphological characteristics (papillae height, papillae width, papillae surface area, and muscular thickness) were recorded in 5 to 8 slides for each sample by using an optical binocular microscope (Olymplus BX 61; Olympus, Warsaw, Poland) connected by a digital camera to the Image-Pro Plus software (version 6.0, Media Cybernetics Inc, Bethesda, MD, USA). For each analyzed parameter, 30 measurements were collected. The muscular thickness of ruminal tissue was determined using a vernier caliper (0 to 150 mm, Chengdu Shuangtuo Testing Instrument Co., Ltd., Chengdu, China) and an analytical balance (Chengdu Shuangtuo Testing Instrument Co., Ltd., Chengdu, China). The papillae height and width were determined by a paraffin slicing machine (Shenzhen Huiwo Technology Co., Ltd., Shenzhen, China) and a scanning electron microscope (Carl Zeiss Management Co., Ltd., Shanghai, China). The cross-sectional papillae surface area was calculated by multiplying papillae height by papillae width (Xiao et al., 2016).

In jejunum, a 2 cm × 2 cm section from mid-portion was placed into 4% paraformaldehyde. The samples were separately cut into 2 cross sections with 1 mm thick, washed, and then embedded in paraffin blocks. For each block, 5 cuts of 3 to 4 µm thick sections were obtained, leading to 10 areas of per site for morphological analysis. The section images were taken and quantitative morphometric analysis was carried out to determine the villus height, villus width, villus surface area, crypt depth, villus height-to-crypt depth ratio, and thickness of the wall by the measurement methods as described above. The cross-sectional villus surface area was calculated by multiplying villus height by villus width, whereas villus height-to-crypt depth ratio was calculated by dividing villus height by crypt depth (Xiao et al., 2016). Additionally, the mucosal samples of jejunal mid-portion were obtained by scratching using a sterile glass microscope slide after washing with ice-cold sterile PBS, and then immediately frozen in liquid nitrogen and stored at −80 °C for qRT-PCR and WB analysis (Zhang et al., 2018).

### Cytokine and TJP gene expressions detected by qRT-PCR

The expression of selected genes, including interleukin-1beta (IL-1β), interleukin-10 (IL-10), tumor necrosis factor-alpha (TNF-α), claudin-1 (CLDN1), occludin (OCLN), and zonula occludens-1 (ZO1), were detected via qRT-PCR. The ruminal epithelia and jejunal mucosal samples of each group were used. Amplification primers of these genes were designed with primer 5.0 software. Total RNA was extracted from samples, and then the cDNA was reversely transcribed from the extracted RNA using cDNA Synthesis Kit (Sangon Biotechnology, Shanghai, China) according to the descriptions. qRT-PCR was carried out by the SYBR Green Kit (Sangon Biotechnology, Shanghai, China) and CFX96 Touch™ Real-Time PCR System (Bio-Rad Inc., Hercules, CA, USA) according to the instructions. Each sample was performed in 3 replicates. GAPDH was used as the housekeeping gene, and the relative expression of genes was calculated using 2^−ΔΔ*Ct*^ method (Livak & Schmittgen, 2001). The primers information of all genes are shown in Table 1.

**Table 1:** Primers used for quantitative real-time PCR.

Genes	GenBank ID	Primer sequence (5′–3′)	Amplicon size, bp
IL-1β	XM_005889988.2	F: GCTTCAGGCAGGTGGTGTCGGTCAT	163
		R: GCGTCACACAGAAACTCGTCGGAGGA	
IL-10	XM_005891650.2	F: GGGTTGCCAAGCCTTGTCGGAAATGA	195
		R: TTCACCTTCTCCACCGCCTTGCTCTT	
TNF-α	XM_005904178.1	F: GCCTCAGCCTCTTCTCCTTCCTCCT	276
		R: GGTTGTCTTCCAGCTTCACACCGTTG	
CLDN1	XM_005897671.2	F: GCATGGAAGACGACGAGGCACAGAAG	196
		R: GCAGCAGCCCAGCCAATGAAGAGAG	
OCLN	XM_005889348.2	F: GCAGCCTCATTACAGCAGCAGTGGTA	161
		R: TGATCCAGTCGTCCTCCAGCTCATCG	
ZO1	XM_014476599.1	F: CGTCCTGATCCTGAGCCTGTGTCTGA	147
		R: ACTCCTCTCCCGACTGGCACTCCTAT	
GAPDH	XM_014482068.1	F: GACTTATGACCACCGTCCACGCCATC	278
		R: CGCCTGCTTCACCACCTTCTTGATCT	

**Notes.**

IL-1β interleukin-1beta IL-10 interleukin-10 TNF- *α* tumor necrosis factor-alpha CLDN1 claudin-1 OCLN occludin ZO-1 zonula occludens-1 GAPDH glyceraldehyde-3-phosphate dehydrogenase F forward R reverse

### TJP expressions detected by WB

Ruminal epithelium and jejunal mucosal samples were homogenized in lysis buffer containing protein inhibitors (Beyotime Biotechnology, Shanghai, China). Total protein was extracted from tissues, and transferred to nitrocellulose membranes (Liu et al., 2013). Immunoblotting was conducted using rabbit anti-CLDN1 (1:2,000 dilution), rabbit anti-OCLN (1:2,000 dilution), and rabbit anti-ZO1 (1:2,000 dilution) antibodies (all from Proteintech). After several washes with Tris Buffered Saline Tween, membranes were incubated with a 1:5,000 dilution of goat anti-rabbit secondary antibody (Thermo Fisher Scientific, Shanghai, China) at room temperature for 2 h. The membranes were incubated with a 1:100,000 dilution of rabbit monoclonal *β*-actin antibody (Thermo Fisher Scientific, Shanghai, China) to normalize the results. Immunoreactivity was determined via chemiluminescence with ECL Plus WB substrate (Thermo Fisher Scientific, Shanghai, China) reference to the instructions and visualized by luminescence imaging (Tanon Technology, Shanghai, China). The density of the blotting band was analyzed by ImageJ software (version 1.46r, National Institutes of Health, USA). Results of each protein are presented as a ratio of densitometry to corresponding *β*-actin value.

### Statistical analysis

Data were shown as mean and standard error of mean (SEM). Before analysis, normality of data were tested first. Then statistical significance of growth performance, serum parameters, morphological parameters, expressions of cytokines (mRNA level), and TJPs (mRNA and protein level) were analyzed by the independent sample *t*-test of the SPSS statistical software (version 19.0 for Windows; SPSS, Chicago, USA), with each yak as an experimental unit. The significance level was indicated at *P* ≤ 0.05, with a trend that was declared at 0.05 < *P* ≤ 0.10. Correlation analysis was done for the mRNA expression levels between cytokines and TJPs, besides, the correlation between ADG and TJPs protein level was analyzed by GraphPad Prism software (version 7.0 for Windows; GraphPad Prism, San Diego, USA). *P* value less than 0.05 and the absolute value of correlation coefficient more than 0.6 were regarded as significant correlation.

## Results

### Growth performance

Growth performance data are presented in Table 2. Obviously, the growth performance of growth-retarded yaks was poor, and the ADG was 0.11 kg/d. The final BW and ADG in GRY group were significantly lower (*P* < 0.05) than those in GNY group.

**Table 2:** The differences of growth performance and serum permeability parameters between growth-retarded and normal yaks.

Items	GNY	GRY	SEM	*P*-value
Growth performance				
Initial BW (kg)	112	74.0	5.03	<0.001
Final BW (kg)	130	80.6	6.79	<0.001
ADG (kg/d)	0.30	0.11	0.03	0.001
Permeability parameters of serum				
DAO (U/L)	19.5	25.9	1.12	0.001
D-LA (mmol/L)	2.98	3.44	0.11	0.035
ET-1 (ng/L)	73.5	69.8	1.99	0.380
LPS (EU/mL)	0.31	0.51	0.04	0.002

**Notes.**

GNY growth normal yaks GRY growth-retarded yaks SEM standard error of mean BW body weight ADG average daily gain DAO diamine oxidase D-LA D-lactic acid ET-1 endotoxin-1 LPS lipopolysaccharide

*n* = 8 for each group.

### Permeability parameters of serum

The differences of permeability parameters in serum between GRY and GNY groups are shown in Table 2. The DAO, D-LA, and LPS concentrations of GNY group exhibited higher (*P* < 0.05) as compared to GNY group. However, no significant difference of ET-1 was observed between 2 groups (*P* > 0.05).

### Morphology of rumen and jejunum

**Figure 1:**
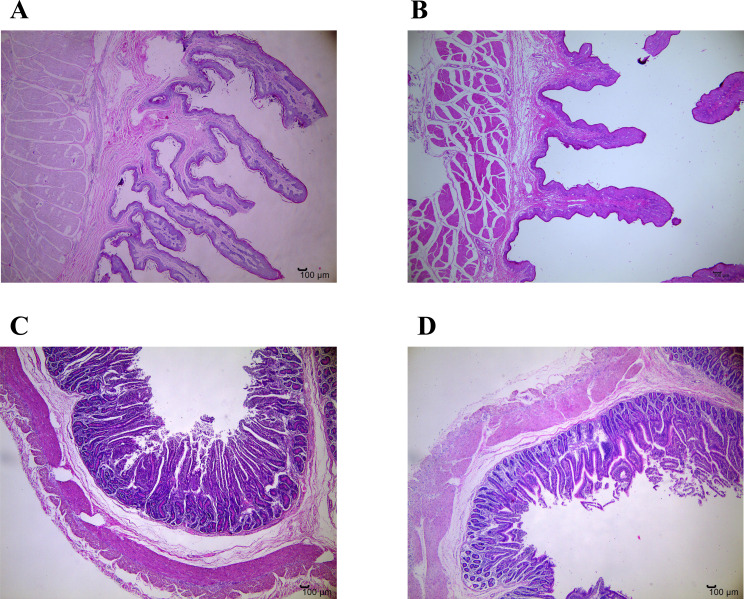
Representative micrographs of ruminal and jejunal epithelium morphology, 40 magnification. (A) Ruminal epithelium of growth normal yak; (B) Ruminal epithelium of growth-retarded yak; (C) Jejunal epithelium of growth normal yak; (D) Jejunal epithelium of growth-retarded yak.

Table 3 and Fig. 1 show the differences of gastrointestinal development between GRY and GNY groups. The ruminal papillae height in GRY group was significantly lower (*P* < 0.05) than that in GNY group. Compared to GNY, the papillae surface area in the rumen of GRY showed a decreasing trend (*P* = 0.063), but no statistical differences of papillae width and muscular thickness were found (*P* > 0.05). In jejunum, with the exception of higher (*P* < 0.05) villus height, width, and surface area in GNY, numerical difference (*P* > 0.05) was detected between 2 groups for crypt depth. However, the villus-to-crypt ratio in GNY group tended to be higher (*P* = 0.069) than that in GRY group.

**Table 3:** The differences of ruminal and jejunal development between growth-retarded and normal yaks.

Items	GNY	GRY	SEM	*P*-value
Rumen				
Papillae height (µm)	1,808	1,331	94.1	0.004
Papillae width (µm)	374	377	22.5	0.950
papillae surface area (µm^2^)	660,773	508,651	41,628	0.063
Muscular thickness (µm)	2,019	1,838	159	0.595
Jejunum				
Villus height (µm)	805	603	39.9	0.004
Villus width (µm)	145	124	5.24	0.038
Villus surface area (µm^2^)	116,572	74,163	7,721	0.001
Crypt depth (µm)	189	206	15.7	0.610
Villus-to-crypt ratio	4.45	3.16	0.36	0.069

**Notes.**

GNY growth normal yaks GRY growth-retarded yaks SEM standard error of mean

*n* = 6 for each group.

### Cytokine mRNA expression of rumen and jejunum

**Figure 2:**
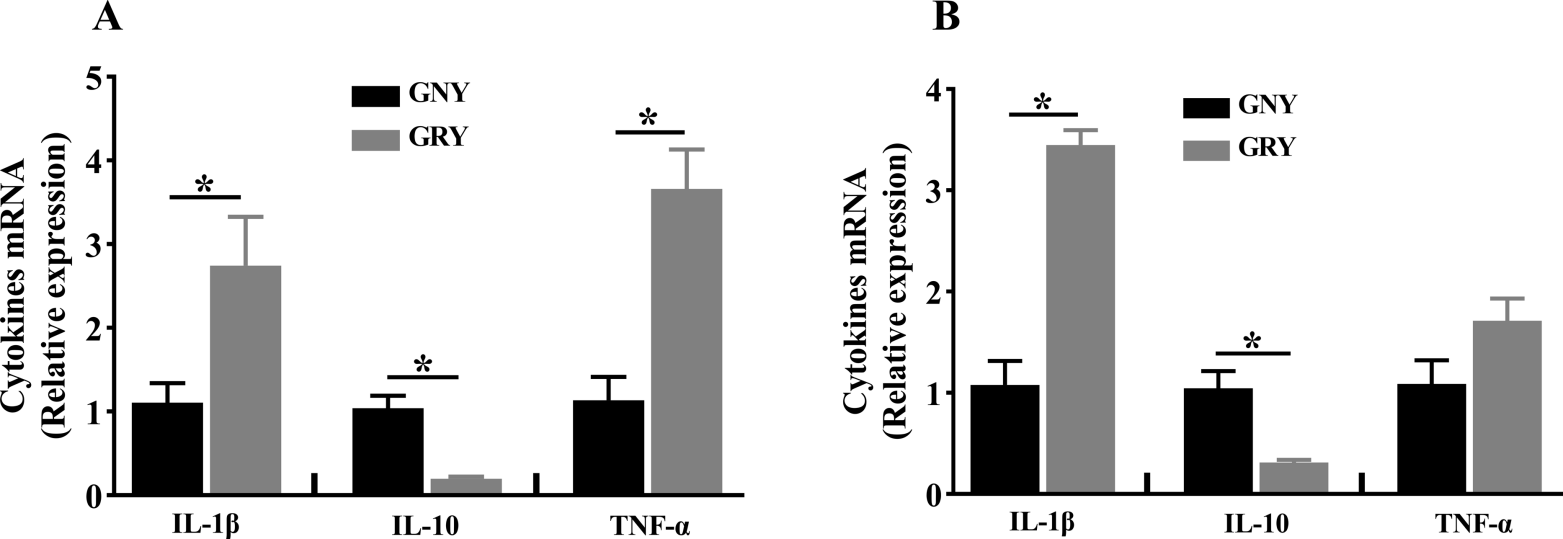
The differences of cytokines mRNA expression levels of rumen (A) and jejunum (B) between growth-retarded and normal yaks. GNY, growth normal yaks; GRY, growth-retarded yaks; IL-1 *β*, interleukin-1beta; IL-10, interleukin-10; TNF-α, tumor necrosis factor-alpha. *n* = 5 for each group. The asterisk (*) indicates a significant difference between two groups (*P* < 0.05).

The mRNA expression levels of cytokines in rumen and jejunum are shown in Fig. 2. Notably, compared to GNY group, the GRY group had higher (*P* < 0.05) IL-1β and TNF-α mRNA expressions, and lower (*P* < 0.05) IL-10 mRNA expression in the rumen (Fig. 2A). A similar trend of IL-1β and IL-10 was found in the jejunum. However, there was no significant difference (*P* > 0.05) of TNF-α mRNA expression in jejunum between 2 groups (Fig. 2B).

### TJP expression of rumen and jejunum

**Figure 3:**
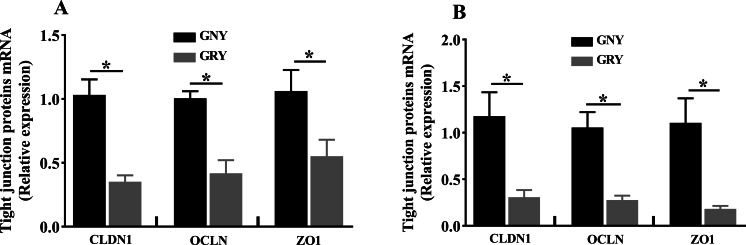
The differences of tight junction proteins mRNA expression levels of rumen (A) and jejunum (B) between growth-retarded and normal yaks. GNY, growth normal yaks; GRY, growth-retarded yaks; CLDN1, claudin-1; OCLN, occludin; ZO1, zonula occludens-1. *n* = 5 for each group. The asterisk (*) indicates a significant difference between two groups (*P* < 0.05).

**Figure 4:**
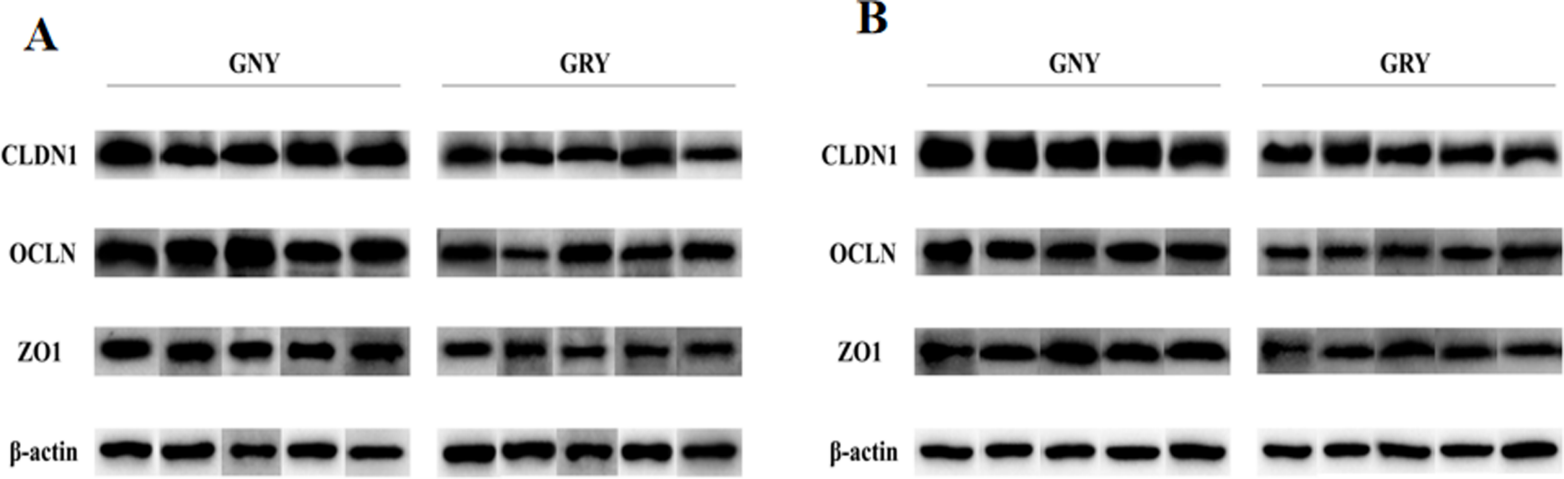
Western blotting analysis of tight junction proteins in rumen (A) and jejunum (B) samples between growth-retarded and normal yaks. Protein extracts of ruminal and jejunal samples were prepared and immunoblotted with specific antibodies. *n* = 5 for each group. GNY, growth normal yaks; GRY, growth-retarded yaks; CLDN1, claudin-1; OCLN, occludin; ZO1, zonula occludens-1.

**Figure 5:**
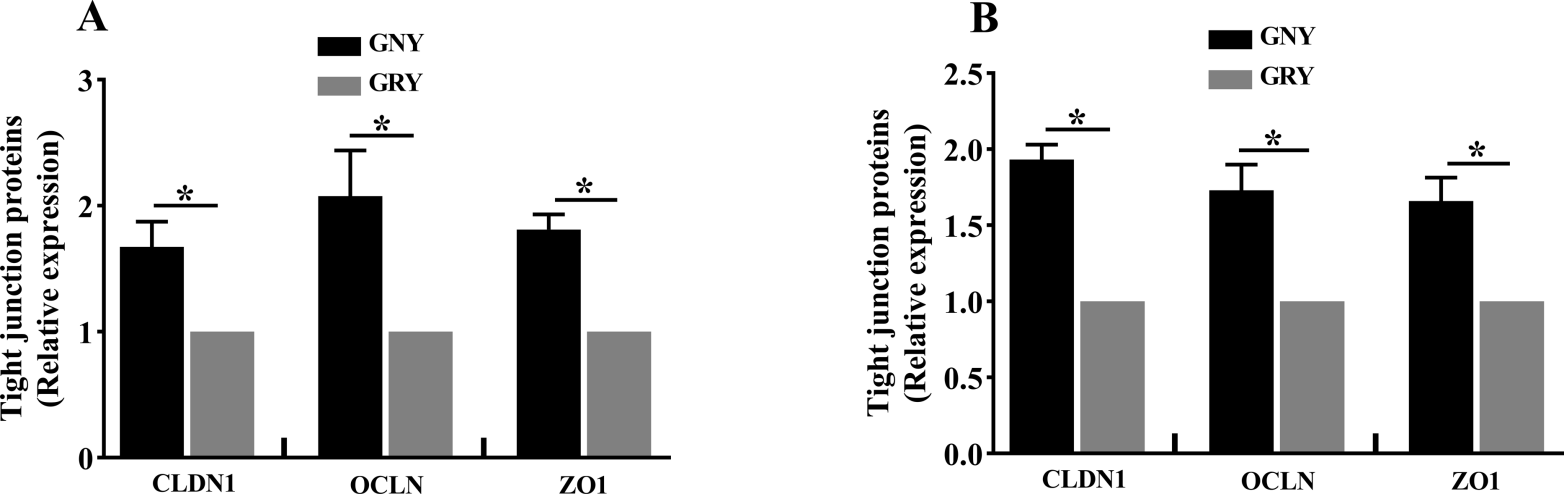
The differences of tight junction proteins expression levels of rumen (A) and jejunum (B) between growth-retarded and normal yaks. GNY, growth normal yaks; GRY, growth-retarded yaks; CLDN1, claudin-1; OCLN, occludin; ZO1, zonula occludens-1. *n* = 5 for each group. The asterisk (*) indicates a significant difference between two groups (*P* < 0.05).

Figure 3 shows the differences of TJPs mRNA expression levels between GRY and GNY groups. Overall, in the rumen (Fig. 3A) and jejunum (Fig. 3B), the GRY group had a decline (*P* < 0.05) in the levels of CLDN1, OCLN, and ZO1 mRNA expressions as compared to GNY group. The protein expression levels of CLDN1, OCLN, and ZO1 were presented in Figs. 4 and 5. Obviously, the rumen (Figs. 4A and 5A) and jejunum (Figs. 4B and 5B) of yaks shared a similar trend. Compared to GNY group, the relative expression levels of all TJPs were lower (*P* < 0.05) in GRY group.

### Correlation analysis of cytokine and TJP

The relationship between cytokines and TJPs mRNA expressions is shown in Table 4. Correlation analysis revealed that there was a positive correlation (*r* > 0.6, *P* < 0.05) between IL-10 mRNA expression and TJPs mRNA expressions in the rumen and jejunum. Conversely, the mRNA expression of TJPs in the rumen and jejunum was negatively correlated (*r* <  − 0.6, *P* < 0.05) with TNF-α mRNA expression. In the jejunum, the relationship between IL-1β and TJPs was similar to TNF-α. However, in the rumen, the mRNA expression of IL-1β was only negatively correlated with OCLN mRNA expression (*r* =  − 0.711, *P* = 0.021, Table 4).

**Table 4:** Correlation analysis between cytokines and tight junction proteins mRNA expressions in the rumen and jejunum.

Items		CLDN1	OCLN	ZO1
Rumen				
IL-1β	*r*-value	−0.596	−0.711^*^	−0.614
	*P*-value	0.069	0.021	0.059
IL-10	*r*-value	0.955^*^	0.779^*^	0.846^*^
	*P*-value	<0.001	0.008	0.002
TNF-α	*r*-value	−0.938^*^	−0.829^*^	−0.907^*^
	*P*-value	<0.001	0.003	<0.001
Jejunum				
IL-1β	*r*-value	−0.828^*^	−0.911^*^	−0.849^*^
	*P*-value	0.003	<0.001	0.002
IL-10	*r*-value	0.781^*^	0.890^*^	0.990^*^
	*P*-value	0.008	0.001	<0.001
TNF-α	*r*-value	−0.763^*^	−0.755^*^	−0.744^*^
	*P*-value	0.010	0.012	0.014

**Notes.**

IL-1β interleukin-1beta IL-10 interleukin-10 TNF- *α* tumor necrosis factor-alpha CLDN1 claudin-1 OCLN occludin ZO1 zonula occludens-1

* *P* < 0.05.

### Correlation analysis of ADG and morphological parameters, TJP expression (protein level)

Table 5 shows the relationship between ADG and gastrointestinal parameters. In rumen, the ADG was positively correlated (*r* = 0.858, *P* = 0.002) with papillae surface area, and a same relationship between ADG and CLDN1, OCLN, and ZO1 protein expressions was found. However, in the jejunum, no significant correlation (*r* = 0.623, *P* = 0.055) was observed between ADG and ZO1 protein expression. There was a positive correlation (*r* > 0.6, *P* < 0.05) between ADG and CLDN1, OCLN proteins expressions.

**Table 5:** Correlation analysis between ADG and morphological parameters, tight junction proteins expressions (protein level) in the rumen and jejunum.

Items		SA	CLDN1	OCLN	ZO1
Rumen					
ADG	*r*-value	0.858^*^	0.751^*^	0.839^*^	0.888^*^
	*P*-value	0.002	0.012	0.002	0.001
Jejunum					
ADG	*r*-value	0.920^*^	0.683^*^	0.844^*^	0.623
	*P*-value	<0.001	0.030	0.002	0.055

**Notes.**

ADG average daily gain SA surface area CLDN1 claudin-1 OCLN occludin ZO1 zonula occludens-1

* *P* < 0.05.

## Discussion

According to our results, there was a positive correlation between ADG and gastrointestinal development. The barrier function has important role in gastrointestinal development. We speculated that the growth retardation of yaks was mainly caused by the functional deficiencies of the gastrointestinal barrier. Therefore, we performed the following study.

### The differences of permeability parameters in serum between growth-retarded and normal yaks

DAO, as a marker of integrity of intestinal mucosa cells, is an intracellular enzyme mainly existing in intestinal epithelial villus of mammalians with high activity (Zhao et al., 2011). Generally, the increased circulating activity of DAO is associated with injured intestinal integrity and barrier function. When the intestinal epithelium is damaged, DAO is released into blood vessels, resulting in elevation of blood DAO (Wang et al., 2016). In the current study, compared to GNY, GRY increased DAO activity in serum, indicating that the intestinal barrier function was impaired. This result was in line with Zhang et al. (2019b), who found that the DAO activity of growth-retarded suckling piglets in serum was higher than normal piglets. The integrity of the gastrointestinal barrier function prevents harmful substances from entering the blood (Albin et al., 2007; Kong et al., 2017). By contrast, previous study reported that the serum content of D-LA in growth-retarded lambs was higher than that in normal lambs (Zhang et al., 2019a). In addition, in ruminant, study has found that goat, which ruminal epithelium was impaired, increased the free LPS in blood (Liu et al., 2013). The results of our experiment were in accordance with above studies, suggesting that the gastrointestinal barrier of growth-retarded yaks exist functional deficiency.

### The differences of gastrointestinal morphology between growth-retarded and normal yaks

Rumen epithelium plays an essential role in the absorption of short-chain fatty acids, and its healthy development has an important effect on nitrogen transportation and urea recycling (Zhao et al., 2018). Ruminal papillae height and width are the most critical factors to evaluate the relative development of rumen epithelium, followed by the thickness of the gastric wall (Lesmeister, Tozer & Heinrichs, 2004). Recent finding showed that growth retardation restricted ruminal development of beef cattle (Du et al., 2018), which was in line with our results. During early age, the normal development of rumen is vital to growth of animals in the future (Xiao et al., 2016). Based on our results, the rumen of yaks with growth retardation was dysplastic. Compared to GRY, GNY had a higher papillae surface area, and this result indicated that the rumen of growth normal yaks had a larger absorption area for short-chain fatty acids and other nutrient ingredients, which was beneficial for growth. Finding an effective additive to improve ruminal development may solve the growth retardation of yaks.

The higher villus height of the small intestine can result in the larger epithelial surface area, and then lead to a stronger ability to digest and absorb nutrients. Besides, deeper crypt depth coinciding with smaller villus-to-crypt ratio can cause nutrient malabsorption (Xiao et al., 2016). Studies in pigs found that growth retardation reduced villus height and surface area of the small intestine (Zhang et al., 2017; Li et al., 2018). In ruminant, the growth-retarded lambs had lower villus height as well as villus-to-crypt ratio (Zhang et al., 2019a). Consistent with these studies, in the present experiment, compared to GRY, GNY exhibited higher villus height, width, and surface area, suggesting that growth normal yaks had stronger ability to absorb nutrients. According to our results, we found that compared to GNY, the gastrointestinal development of GRY was dysplastic. In the future, more studies should focus on how to promote gastrointestinal development of yaks.

### The differences of ruminal barrier function between growth-retarded and normal yaks

As a component of the immune system, the ruminal epithelial barrier is critical for ruminants to absorb nutrients and maintain healthy growth (Penner et al., 2011). The tight junction, which is located at the top of the epithelial cells, plays an important role to prevent harmful pathogenic bacteria from entering the submucosa through the epithelial space, besides, the tight junction is useful to regulate the permeability of the epithelium and selectively transport nutrients (Greco et al., 2018). Compared to GNY, the data from our study demonstrated that the CLDN1, OCLN, and ZO1 expression at mRNA and protein levels in GRY were down-regulated. There are several possible reasons for changes in TJPs. On the one hand, the changes in TJPs may be associated with the dysplastic structure of the ruminal epithelium. Study have shown that decreased granulosum stratum thickness of ruminal epithelium could down-regulate expressions of TJPs (Liu et al., 2013). The ultrastructure of growth-retarded yaks’ ruminal epithelium needs further evaluation. On the other hand, high LPS concentration may affect TJPs expressions. Study in goat has demonstrated that higher LPS concentration reduced the expressions of ruminal TJPs (Zhang et al., 2019c). Nevertheless, the mechanisms behind the difference in TJPs between GNY and GRY need to be further elucidation.

Local inflammation in the rumen commonly affects its healthy development (Plaizier et al., 2012). Our study investigated the relationship between inflammation and TJPs in the rumen. In the present study, the mRNA expressions of pro-inflammatory cytokines IL-1β and TNF-α were elevated in the ruminal epithelium of growth-retarded yaks, and TNF-α was negatively correlated with TJPs expressions. These results indicated that GRY-induced local inflammation in the rumen was related to the changes of proteins expressions. Liu et al. (2013) found that the disruption expression of TJPs in ruminal epithelium caused an increase of ruminal epithelial permeability, and resulted in bacteria translocation, then induced inflammatory responses of rumen, which were in line with our results. Additionally, the cytokines have been demonstrated to play an essential role in the regulation of epithelial barrier function via changing TJPs expressions (Mazzon & Cuzzocrea, 2008). It is possible that the up-regulated IL-1 *β* and TNF-α could alter the TJPs expressions of ruminal epithelium in GRY. In turn, based on our data, we can detect the cytokines to evaluate the ruminal barrier junction of yaks.

### The differences of jejunal barrier function between growth-retarded and normal yaks

As the first line of limiting the invasion of toxins, microbes, and food antigens, the intestinal epithelial barrier has a vital role in the healthy growth of animals (Yi et al., 2018). Previous study found that the OCLN protein expression of jejunum in growth-retarded piglets was lower than growth normal piglets (Su et al., 2017). Consistent with that study, our experiment found that compared to GNY, the OCLN protein expression in GRY was down-regulated. In addition, we also found GRY exhibited lower expressions of CLDN1 and ZO1 both in mRNA and protein levels, suggesting that there was a functional deficiency in the jejunal epithelium. Recent findings have shown that many factors, such as weaning stress and malnutrition, could cause the intestinal dysfunction, besides, the disruption of intestinal barrier function could affect animals’ normal growth (Xing et al., 2017; Song et al., 2017). Another study found that TJPs depletion in intestinal epithelium led to a selective increase of macromolecules, including LPS, in blood (Al-Sadi et al., 2011), which was in accordance with the result of serum permeability parameters. In our study, it is likely that malnutrition during early age of GRY causes intestinal barrier dysfunction, and then results in growth retardation.

Consistent with rumen, the balance of pro-inflammatory and anti-inflammatory cytokines is of great importance for the health of animals, and also associated with the intestinal function (Hu et al., 2018). Previous study reported that the jejunal mRNA expressions of pro-inflammatory cytokines, such as IL-1β and TNF- *α*, were up-regulated in growth-retarded piglets (Zhang et al., 2019b). Consistent with previous study, our experiment found that the IL-1β and TNF-α mRNA expressions in jejunum of GRY were higher than those of GNY. Moreover, compared to GNY, the mRNA expression of anti-inflammatory cytokine IL-10 in GRY was lower. The data indicated that the jejunum had inflammation. Additionally, in the present study, the cytokines correlated (positively, IL-10; negatively, IL-1β and TNF-α) with TJPs expressions. The results were in accordance with John, Fromm & Schulzke (2011), who found that the cytokines could regulate the expressions of TJPs. According to all the data of the current study, it is suggested that the breakdown of gastrointestinal barrier involving both the higher expressions of pro-inflammatory cytokines and disruption of TJPs potentially increases the digestive epithelial permeability, providing opportunity for translocation of the harmful substances from the digestive tract into blood circulation. Moreover, previous studies have reported that some additives, including glutamine, *Bacillus subtilis*, and plant bioactive compounds, can be used to improve the gastrointestinal barrier function of animals (Wu et al., 2018; Tang et al., 2019; Patra, Amasheh & Aschenbach, 2019). These additives can increase the TJPs expressions, regulate the cytokines secretion, and decrease permeability of the gastrointestinal tract. The compensatory growth of animals is a common phenomenon, and an effective solution to promote compensatory growth of GRY is required. In the future, we will do more studies about how to promote compensatory growth of GRY with effective additive.

## Conclusions

The results of our study indicated that compared to growth normal yaks, the ruminal and jejunal barrier function of growth-retarded yaks are disrupted. Based on our study, finding an additive (e.g., glutamine, *Bacillus subtilis*, and plant bioactive compounds) that can improve gastrointestinal barrier function may be a potential solution to promote growth of growth-retarded yaks.

##  Supplemental Information

10.7717/peerj.9851/supp-1 Supplemental Information 1 Supplemental Tables

10.7717/peerj.9851/supp-2 Supplemental Information 2 Blots

## Additional Information and Declarations

### Data Availability

The following information was supplied regarding data availability: All data generated or analyzed in the current study are available from the Supplemental Files.
