# Supplementary material for: Comparing the gastrointestinal barrier function between growth-retarded and normal yaks on the Qinghai-Tibetan Plateau
Source: PeerJ. 2020 Sep 3;8:e9851. doi: 10.7717/peerj.9851 (PMC7474896; doi:10.7717/peerj.9851)

**Comparing the gastrointestinal barrier function between growth-retarded and normal yaks on the Qinghai-Tibetan Plateau**

Jian Ma^1^, Ali Mujtaba Shah^1^, Zhi-Sheng Wang^1^, Rui Hu^1^, Hua-Wei Zou, Xue-Ying Wang^1^, Guang Cao^1^, Quan-Hui Peng^1^, Bai Xue^1^, Li-Zhi Wang^1^, Suo-Nan Zhao^2^ and Xiang-Ying Kong^2^

^1^ Low Carbon Breeding Cattle and Safety Production University Key Laboratory of Sichuan Province, Animal Nutrition Institute, Sichuan Agricultural University, Chengdu, Sichuan, P.R. China

^2^ Haibei Demonstration Zone of Plateau Modern Ecological Animal Husbandry Science and Technology, Haibei, Qinghai, P.R. China

1. Growth performance

|  | Animals No. | Initial BW (kg) | Final BW (kg) | ADG (kg) |
| --- | --- | --- | --- | --- |
| GNY | 0502 | 110 | 123 | 0.216666667 |
|  | 0689 | 107 | 121 | 0.233333333 |
|  | 0680 | 112 | 132 | 0.333333333 |
|  | 0690 | 119 | 154 | 0.583333333 |
|  | 0679 | 107 | 123 | 0.266666667 |
|  | 0691 | 114 | 132 | 0.3 |
|  | 0681 | 110 | 125 | 0.25 |
|  | 0682 | 114 | 128 | 0.233333333 |
| GRY | 0711 | 74 | 80 | 0.1 |
|  | 0697 | 81 | 94 | 0.216666667 |
|  | 0699 | 85 | 93 | 0.133333333 |
|  | 0693 | 67 | 70 | 0.05 |
|  | 0708 | 72 | 75 | 0.05 |
|  | 0686 | 73 | 85 | 0.2 |
|  | 0731 | 74 | 78 | 0.066666667 |
|  | 0884 | 66 | 70 | 0.066666667 |

GNY, growth normal yaks; GRY, growth-retarded yaks; BW, body weight; ADG, average daily gain.

2. Permeability parameters in serum

|  | Animals No. | DAO (U/L) | D-LA (mmol/L) | ET-1 (ng/L) | LPS (EU/mL) |
| --- | --- | --- | --- | --- | --- |
| GNY | 0502 | 15.69 | 2.68 | 54.67 | 0.19 |
|  | 0689 | 26.29 | 3.37 | 83.56 | 0.53 |
|  | 0680 | 20.21 | 2.79 | 70.04 | 0.35 |
|  | 0690 | 18.36 | 3.05 | 74.18 | 0.28 |
|  | 0679 | 17.04 | 2.33 | 75.72 | 0.30 |
|  | 0691 | 21.09 | 3.01 | 73.36 | 0.26 |
|  | 0681 | 18.11 | 3.27 | 78.43 | 0.29 |
|  | 0682 | 19.02 | 3.30 | 77.71 | 0.31 |
| GRY | 0711 | 20.06 | 2.88 | 58.54 | 0.33 |
|  | 0697 | 29.53 | 4.07 | 82.45 | 0.70 |
|  | 0699 | 27.55 | 3.68 | 70.04 | 0.42 |
|  | 0693 | 23.86 | 3.26 | 65.78 | 0.65 |
|  | 0708 | 26.01 | 3.84 | 72.56 | 0.66 |
|  | 0686 | 24.48 | 3.01 | 76.65 | 0.36 |
|  | 0731 | 27.41 | 3.61 | 67.04 | 0.46 |
|  | 0884 | 28.02 | 3.13 | 65.56 | 0.53 |

GNY, growth normal yaks; GRY, growth-retarded yaks; DAO, diamine oxidase; D-LA, D-lactic acid; ET-1, endotoxin-1; LPS, lipopolysaccharide.

3. Ruminal morphology

|  | Animals No. | PH (μm) | PW (μm) | PSA (μm^2^) | MT (μm) |
| --- | --- | --- | --- | --- | --- |
| GNY | 0689 | 2021.75 | 301.32 | 609193.71 | 2801.17 |
|  | 0680 | 1588.53 | 402.33 | 639113.2749 | 1896.83 |
|  | 0690 | 1898.44 | 301.88 | 573101.0672 | 1205.9 |
|  | 0691 | 1709.64 | 379.29 | 648449.3556 | 2360.84 |
|  | 0681 | 1525.05 | 546.54 | 833500.827 | 2390.16 |
|  | 0682 | 2106.93 | 313.86 | 661281.0498 | 1459.37 |
| GRY | 0711 | 1548.68 | 413.87 | 640952.1916 | 2329.63 |
|  | 0697 | 1102.34 | 422.31 | 465529.2054 | 1109.89 |
|  | 0699 | 1268.85 | 331.44 | 420547.644 | 1889.49 |
|  | 0693 | 1215.2 | 325.4 | 395426.08 | 1643.87 |
|  | 0708 | 1612.53 | 468.11 | 754841.4183 | 2529.1 |
|  | 0686 | 1239.08 | 302.33 | 374611.0564 | 1529.93 |

GNY, growth normal yaks; GRY, growth-retarded yaks; PH, papillae height; PW, papillae width; PSA, papillae surface area; MT, muscular thickness.

4. Jejunal morphology

|  | Animals No. | VH (μm) | VW (μm) | VSA (μm^2^) | CD (μm) | VCR |
| --- | --- | --- | --- | --- | --- | --- |
| GNY | 0689 | 952.39 | 140.03 | 133363.1717 | 202.31 | 4.70757748 |
|  | 0680 | 862.39 | 136.73 | 117914.5847 | 160.36 | 5.377837366 |
|  | 0690 | 818.34 | 172.69 | 141319.1346 | 144.39 | 5.667567006 |
|  | 0691 | 734.43 | 152.21 | 111787.5903 | 233.64 | 3.143425783 |
|  | 0681 | 682.39 | 130.72 | 89202.0208 | 146.19 | 4.667829537 |
|  | 0682 | 779.22 | 135.84 | 105849.2448 | 249.4 | 3.124378508 |
| GRY | 0711 | 599.68 | 118.38 | 70990.1184 | 301.96 | 1.985958405 |
|  | 0697 | 509.25 | 133.06 | 67760.805 | 202.69 | 2.512457447 |
|  | 0699 | 665.69 | 117.09 | 77945.6421 | 131.71 | 5.054210007 |
|  | 0693 | 641.57 | 147.09 | 94368.5313 | 166.69 | 3.848881157 |
|  | 0708 | 482.39 | 123.13 | 59396.6807 | 169.5 | 2.845958702 |
|  | 0686 | 717.93 | 103.80 | 74521.134 | 266.38 | 2.69513477 |

GNY, growth normal yaks; GRY, growth-retarded yaks; VH, villus height; VW, villus width; VSA, villus surface area; CD, crypt depth; VCR, villus-to-crypt ratio.

5. mRNA expression of cytokines in rumen and jejunum

|  | Animals NO. | Rumen | | | Jejunum | | |
| --- | --- | --- | --- | --- | --- | --- | --- |
|  |  | IL-1β | IL-10 | TNF-α | IL-1β | IL-10 | TNF-α |
| GNY | 0689 | 0.56 | 0.90 | 1.82 | 0.77 | 1.02 | 0.68 |
|  | 0680 | 1.53 | 0.70 | 1.78 | 0.84 | 1.66 | 0.68 |
|  | 0690 | 1.67 | 1.56 | 0.91 | 2.01 | 0.71 | 1.36 |
|  | 0691 | 1.19 | 1.16 | 0.56 | 1.00 | 0.78 | 1.88 |
|  | 0682 | 0.58 | 0.87 | 0.61 | 0.77 | 1.06 | 0.84 |
| GRY | 0711 | 1.57 | 0.22 | 4.25 | 3.48 | 0.27 | 1.97 |
|  | 0697 | 4.07 | 0.16 | 3.56 | 3.03 | 0.23 | 0.98 |
|  | 0699 | 3.97 | 0.19 | 2.51 | 3.94 | 0.31 | 1.44 |
|  | 0693 | 1.25 | 0.14 | 2.87 | 3.36 | 0.36 | 2.17 |
|  | 0708 | 2.85 | 0.28 | 5.11 | 3.43 | 0.38 | 2.00 |

GNY, growth normal yaks; GRY, growth-retarded yaks; IL-1β, interleukin-1beta; IL-10, interleukin-10; TNF-α, tumor necrosis factor-alpha.

6. mRNA expression of tight junction proteins in rumen and jejunum

|  | Animals NO. | Rumen | | | Jejunum | | |
| --- | --- | --- | --- | --- | --- | --- | --- |
|  |  | CLDN1 | OCLN | ZO1 | CLDN1 | OCLN | ZO1 |
| GNY | 0689 | 0.64 | 1.17 | 0.51 | 0.80 | 1.49 | 1.04 |
|  | 0680 | 1.26 | 1.06 | 1.23 | 0.81 | 1.29 | 2.10 |
|  | 0690 | 1.25 | 0.93 | 1.20 | 2.09 | 0.58 | 0.56 |
|  | 0691 | 1.14 | 1.02 | 1.47 | 1.40 | 0.79 | 0.81 |
|  | 0682 | 0.87 | 0.85 | 0.90 | 0.79 | 1.13 | 1.02 |
| GRY | 0711 | 0.23 | 0.19 | 0.30 | 0.43 | 0.15 | 0.18 |
|  | 0697 | 0.39 | 0.72 | 0.85 | 0.28 | 0.34 | 0.09 |
|  | 0699 | 0.29 | 0.57 | 0.79 | 0.48 | 0.41 | 0.14 |
|  | 0693 | 0.33 | 0.21 | 0.21 | 0.31 | 0.20 | 0.24 |
|  | 0708 | 0.52 | 0.40 | 0.61 | 0.04 | 0.29 | 0.26 |

GNY, growth normal yaks; GRY, growth-retarded yaks; CLDN1, claudin-1; OCLN, occludin; ZO1, zonula occludens-1.

7. Western blotting analysis of tight junction proteins (A: CLDN1; B: OCLN; C: ZO1) in ruminal samples between growth-retarded and normal yaks. 1 represents growth-retarded yaks (GRY); 2 represents growth normal yaks (GNY). CLDN1, claudin-1; OCLN, occludin; ZO1, zonula occludens-1.


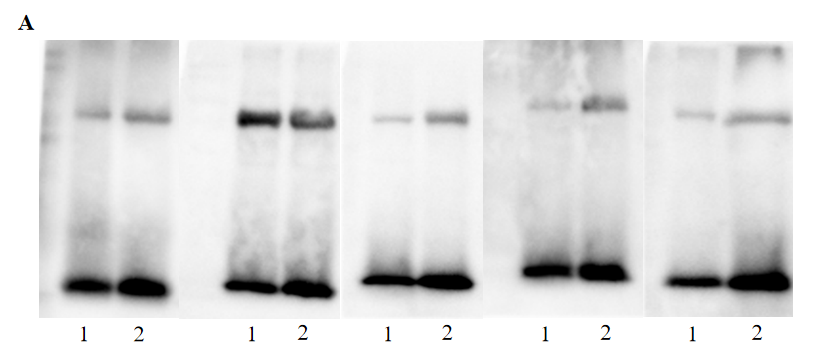


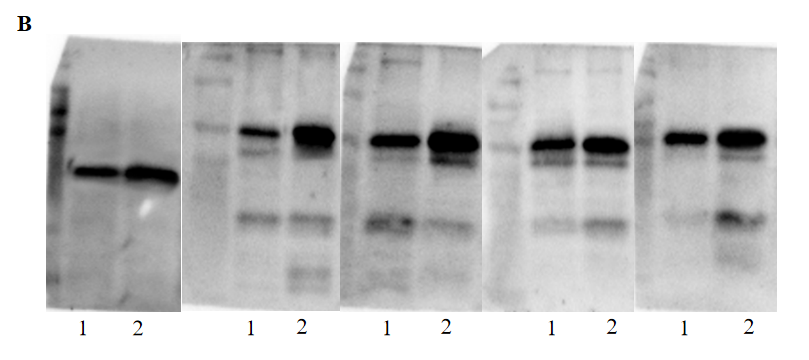


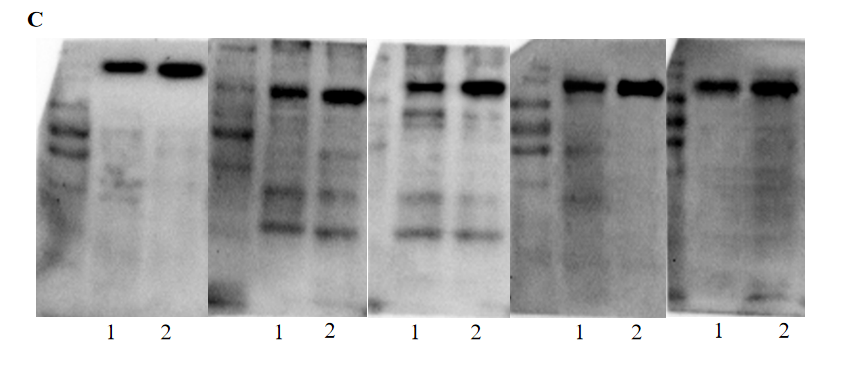


8. Western blotting analysis of tight junction proteins (A: CLDN1; B: OCLN; C: ZO1) in jejunal samples between growth-retarded and normal yaks. 1 represents growth-retarded yaks (GRY); 2 represents growth normal yaks (GNY). CLDN1, claudin-1; OCLN, occludin; ZO1, zonula occludens-1.


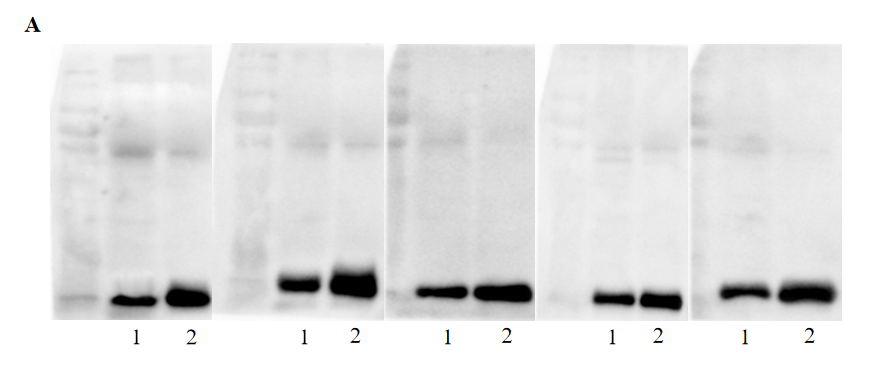


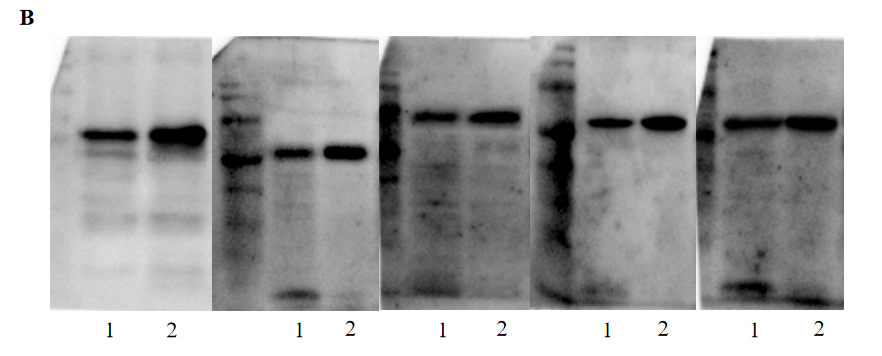


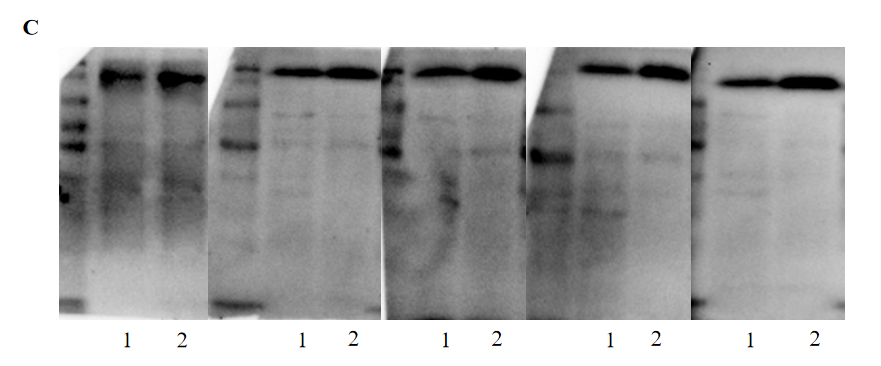

Supplement: Supplemental Information 1 [file peerj-08-9851-s001.doc]
