# Supplementary figures and images for: Comparing the gastrointestinal barrier function between growth-retarded and normal yaks on the Qinghai-Tibetan Plateau
Source: PeerJ. 2020 Sep 3;8:e9851. doi: 10.7717/peerj.9851 (PMC7474896; doi:10.7717/peerj.9851)

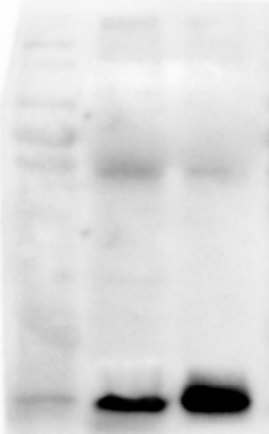

Supplement: Supplemental Information 2 [file peerj-08-9851-s002.zip › Supplementary materials-blots/CLDN1-Jejunum/1.png]

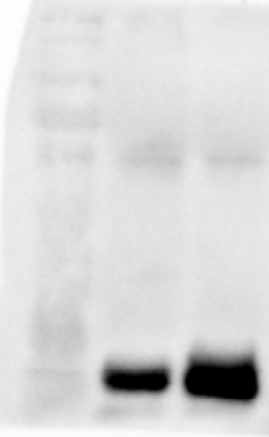

Supplement: Supplemental Information 2 [file peerj-08-9851-s002.zip › Supplementary materials-blots/CLDN1-Jejunum/2.png]

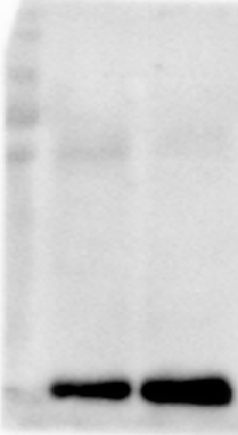

Supplement: Supplemental Information 2 [file peerj-08-9851-s002.zip › Supplementary materials-blots/CLDN1-Jejunum/3.png]

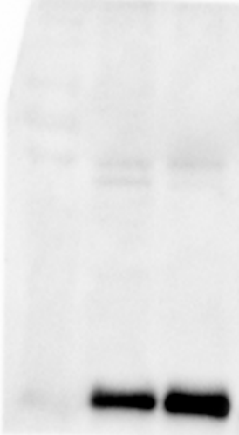

Supplement: Supplemental Information 2 [file peerj-08-9851-s002.zip › Supplementary materials-blots/CLDN1-Jejunum/4.png]

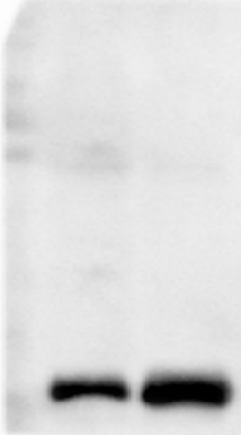

Supplement: Supplemental Information 2 [file peerj-08-9851-s002.zip › Supplementary materials-blots/CLDN1-Jejunum/5.png]

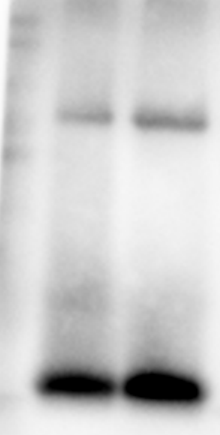

Supplement: Supplemental Information 2 [file peerj-08-9851-s002.zip › Supplementary materials-blots/CLDN1-Rumen/1.png]

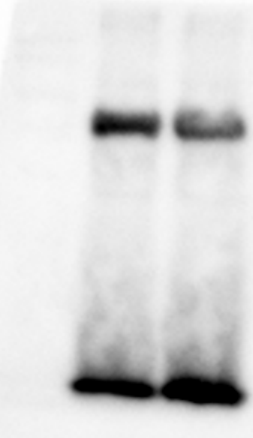

Supplement: Supplemental Information 2 [file peerj-08-9851-s002.zip › Supplementary materials-blots/CLDN1-Rumen/2.png]

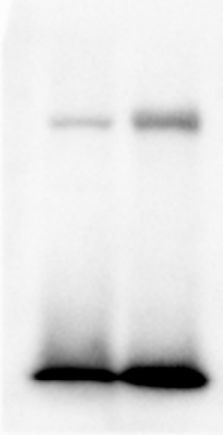

Supplement: Supplemental Information 2 [file peerj-08-9851-s002.zip › Supplementary materials-blots/CLDN1-Rumen/3.png]

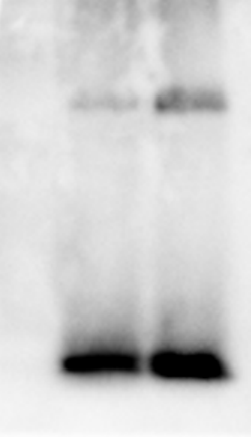

Supplement: Supplemental Information 2 [file peerj-08-9851-s002.zip › Supplementary materials-blots/CLDN1-Rumen/4.png]

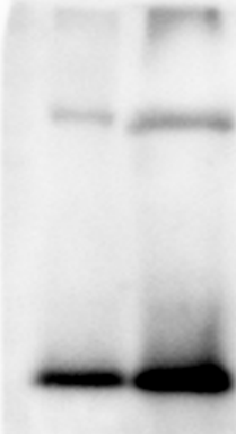

Supplement: Supplemental Information 2 [file peerj-08-9851-s002.zip › Supplementary materials-blots/CLDN1-Rumen/5.png]

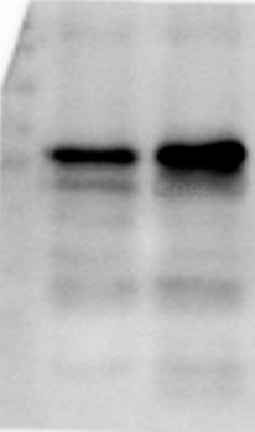

Supplement: Supplemental Information 2 [file peerj-08-9851-s002.zip › Supplementary materials-blots/OCLN-Jejunum/1.png]

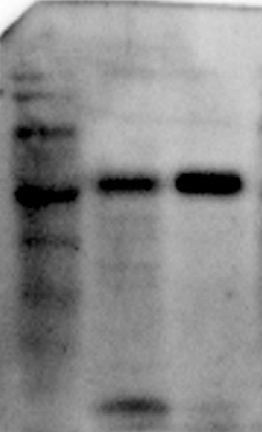

Supplement: Supplemental Information 2 [file peerj-08-9851-s002.zip › Supplementary materials-blots/OCLN-Jejunum/2.png]

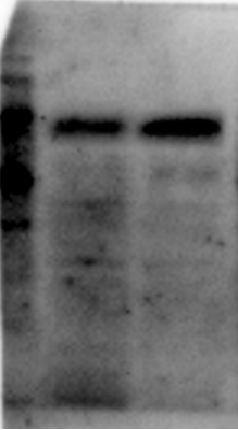

Supplement: Supplemental Information 2 [file peerj-08-9851-s002.zip › Supplementary materials-blots/OCLN-Jejunum/3.png]

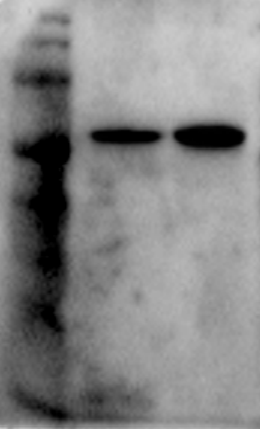

Supplement: Supplemental Information 2 [file peerj-08-9851-s002.zip › Supplementary materials-blots/OCLN-Jejunum/4.png]

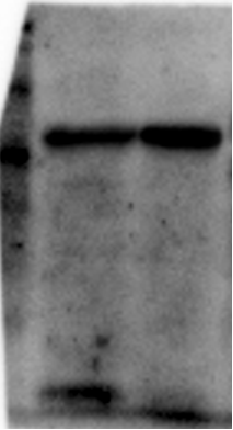

Supplement: Supplemental Information 2 [file peerj-08-9851-s002.zip › Supplementary materials-blots/OCLN-Jejunum/5.png]

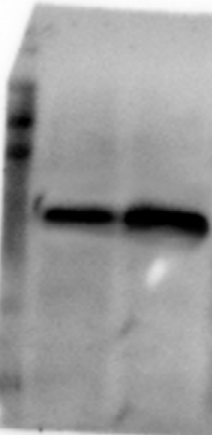

Supplement: Supplemental Information 2 [file peerj-08-9851-s002.zip › Supplementary materials-blots/OCLN-Rumen/1.png]

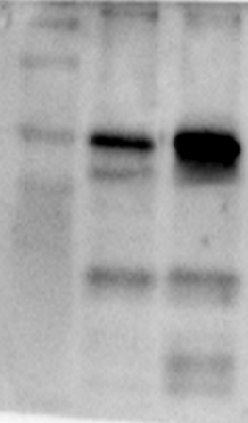

Supplement: Supplemental Information 2 [file peerj-08-9851-s002.zip › Supplementary materials-blots/OCLN-Rumen/2.png]

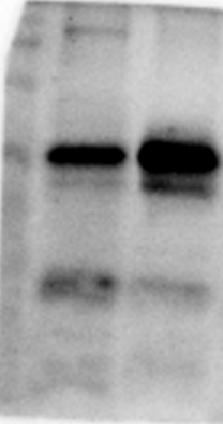

Supplement: Supplemental Information 2 [file peerj-08-9851-s002.zip › Supplementary materials-blots/OCLN-Rumen/3.png]

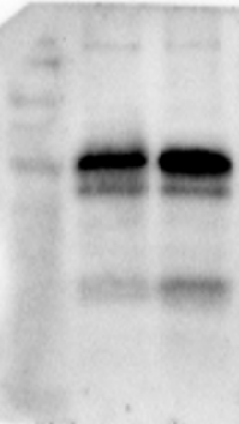

Supplement: Supplemental Information 2 [file peerj-08-9851-s002.zip › Supplementary materials-blots/OCLN-Rumen/4.png]

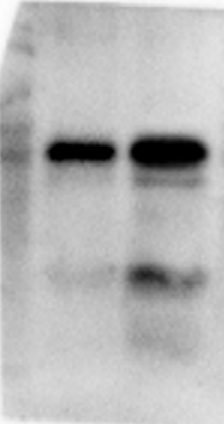

Supplement: Supplemental Information 2 [file peerj-08-9851-s002.zip › Supplementary materials-blots/OCLN-Rumen/5.png]

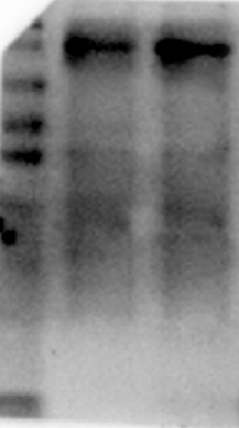

Supplement: Supplemental Information 2 [file peerj-08-9851-s002.zip › Supplementary materials-blots/ZO1-Jejunum/1.png]

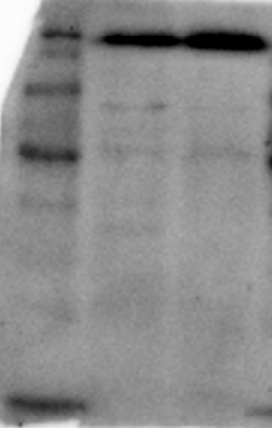

Supplement: Supplemental Information 2 [file peerj-08-9851-s002.zip › Supplementary materials-blots/ZO1-Jejunum/2.png]

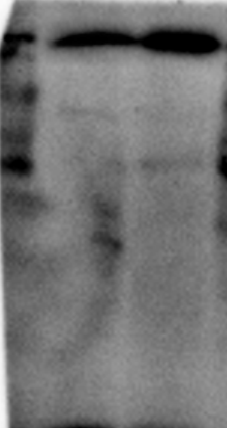

Supplement: Supplemental Information 2 [file peerj-08-9851-s002.zip › Supplementary materials-blots/ZO1-Jejunum/3.png]

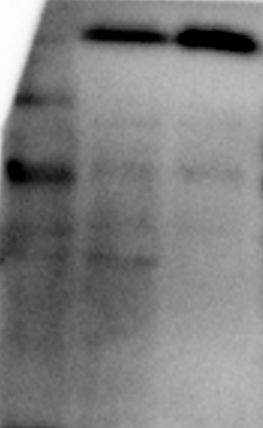

Supplement: Supplemental Information 2 [file peerj-08-9851-s002.zip › Supplementary materials-blots/ZO1-Jejunum/4.png]

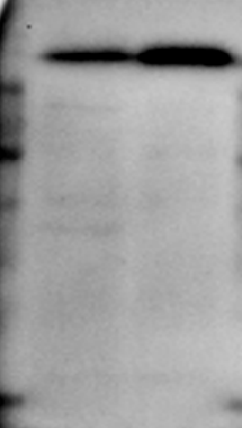

Supplement: Supplemental Information 2 [file peerj-08-9851-s002.zip › Supplementary materials-blots/ZO1-Jejunum/5.png]

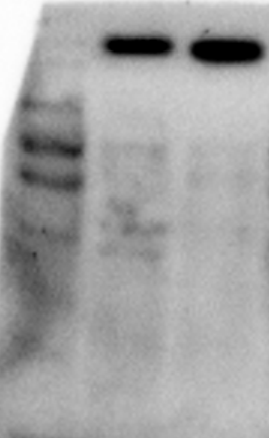

Supplement: Supplemental Information 2 [file peerj-08-9851-s002.zip › Supplementary materials-blots/ZO1-Rumen/1.png]

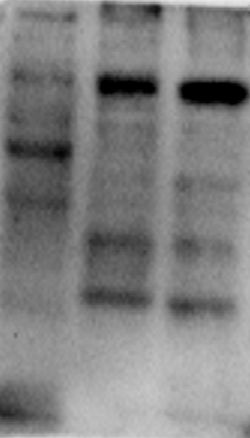

Supplement: Supplemental Information 2 [file peerj-08-9851-s002.zip › Supplementary materials-blots/ZO1-Rumen/2.png]

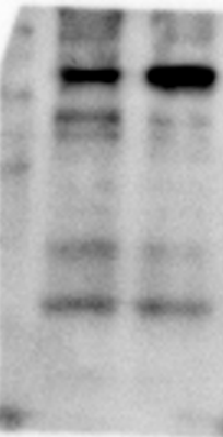

Supplement: Supplemental Information 2 [file peerj-08-9851-s002.zip › Supplementary materials-blots/ZO1-Rumen/3.png]

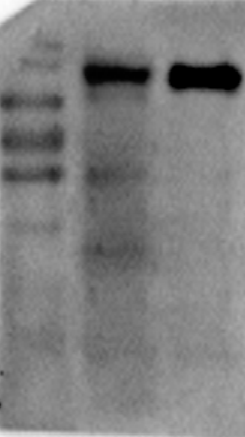

Supplement: Supplemental Information 2 [file peerj-08-9851-s002.zip › Supplementary materials-blots/ZO1-Rumen/4.png]

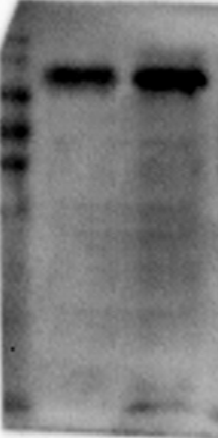

Supplement: Supplemental Information 2 [file peerj-08-9851-s002.zip › Supplementary materials-blots/ZO1-Rumen/5.png]

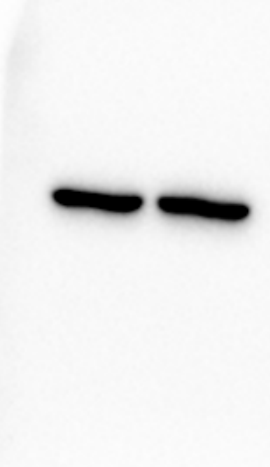

Supplement: Supplemental Information 2 [file peerj-08-9851-s002.zip › Supplementary materials-blots/a┬-actin-Jejunum/1.png]

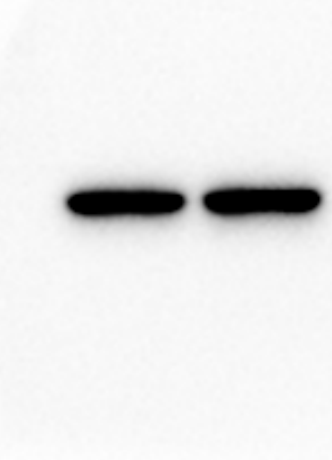

Supplement: Supplemental Information 2 [file peerj-08-9851-s002.zip › Supplementary materials-blots/a┬-actin-Jejunum/2.png]

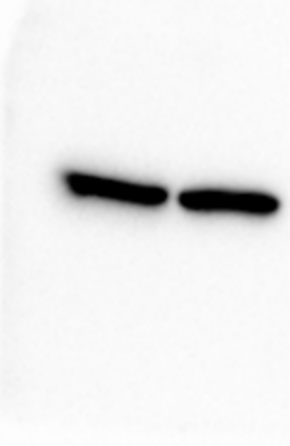

Supplement: Supplemental Information 2 [file peerj-08-9851-s002.zip › Supplementary materials-blots/a┬-actin-Jejunum/3.png]

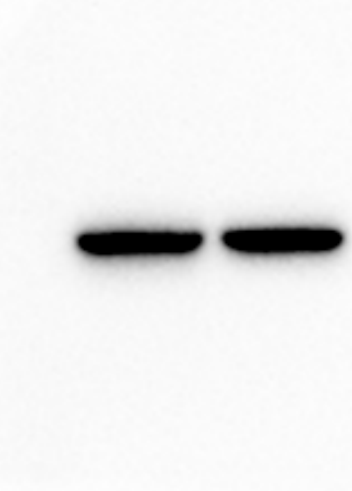

Supplement: Supplemental Information 2 [file peerj-08-9851-s002.zip › Supplementary materials-blots/a┬-actin-Jejunum/4.png]

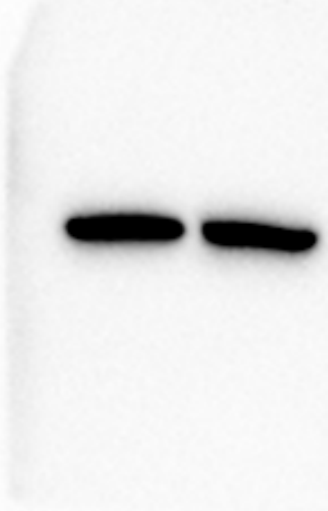

Supplement: Supplemental Information 2 [file peerj-08-9851-s002.zip › Supplementary materials-blots/a┬-actin-Jejunum/5.png]

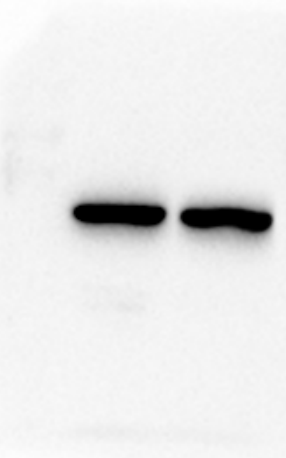

Supplement: Supplemental Information 2 [file peerj-08-9851-s002.zip › Supplementary materials-blots/a┬-actin-Rumen/1.png]

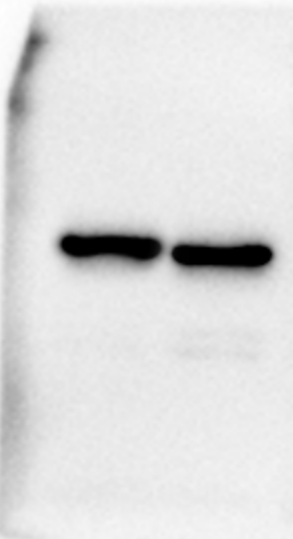

Supplement: Supplemental Information 2 [file peerj-08-9851-s002.zip › Supplementary materials-blots/a┬-actin-Rumen/2.png]

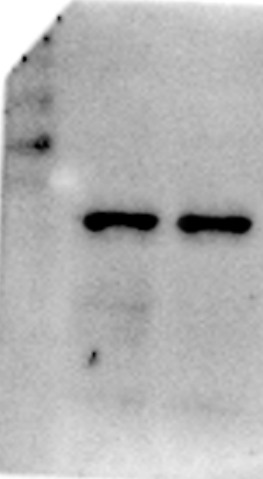

Supplement: Supplemental Information 2 [file peerj-08-9851-s002.zip › Supplementary materials-blots/a┬-actin-Rumen/3.png]

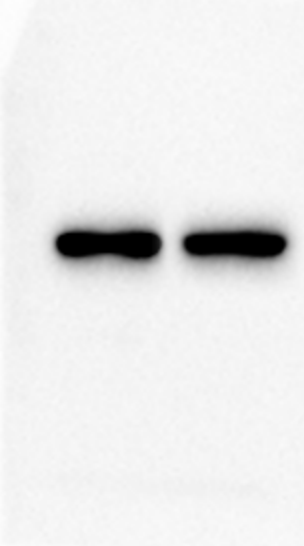

Supplement: Supplemental Information 2 [file peerj-08-9851-s002.zip › Supplementary materials-blots/a┬-actin-Rumen/4.png]

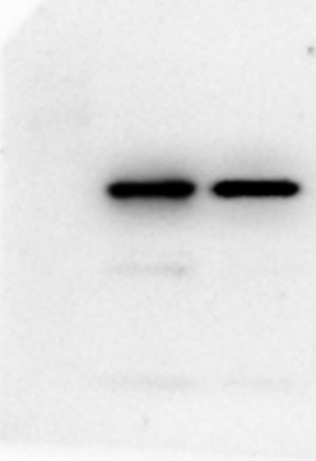

Supplement: Supplemental Information 2 [file peerj-08-9851-s002.zip › Supplementary materials-blots/a┬-actin-Rumen/5.png]
